# Soil pH and Nitrogen Content Drive the Succession of RubisCO-Harboring Microbial Communities Across *Picea asperata* Plantation Ages

**DOI:** 10.3390/biology15090725

**Published:** 2026-05-02

**Authors:** Dehui Li, Yaodan Deng, Xiaohui Zhao, Qian Liao, Jialing Chen, Chaonan Li, Haijun Liao

**Affiliations:** 1 School of Human Settlements, Mianyang Teachers’ College, Mianyang 621000, China; dehli@mtc.edu.cn (D.L.); jinxo733466@163.com (Y.D.); zhaoxh69@163.com (X.Z.); lq20020619@163.com (Q.L.); jialing212@163.com (J.C.); 2 Sichuan Provincial Forestry and Grassland Engineering Research Center of Ancient and Notable Trees Protection, The Forestry and Grassland Bureau of Sichuan Province, Chengdu 610081, China; 3 School of Life Sciences (School of Ecological Forestry), Mianyang Teachers’ College, Mianyang 621000, China; licn@mtc.edu.cn

**Keywords:** carbon-fixing microbes, RubisCO, Calvin–Benson–Bassham cycle, stand age, *Picea asperata* plantation

## Abstract

Autotrophic carbon-fixing microbes can assimilate atmospheric carbon dioxide into biomass via the Calvin–Benson–Bassham (CBB) cycle (their primary carbon fixation pathway), thereby reinforcing soil carbon sequestration in the plantation ecosystem; however, the succession of RubisCO-harboring microbial communities across stand ages remains poorly understood. Here, we investigated the community succession of microbes carrying the gene encoding RubisCO, a key enzyme in the CBB cycle, across a stand-age chronosequence in a *Picea asperata* plantation ecosystem. Our results revealed a progressive decrease in microbial α-diversity and a significant restructuring of community composition with increasing stand age, characterized by an enrichment of *Proteobacteria* and a concomitant depletion of *Actinobacteria*. While the Shannon–Wiener index was most strongly correlated with soil total nitrogen content, redundancy analysis identified soil pH as the predominant environmental driver of community turnover, a relationship that was found to be threshold-dependent, with substantial community shifts occurring in response to pH variations of 0.5 to 1.0 units. These findings suggest that sustaining the diversity of RubisCO-harboring microbes in older stands—a process potentially enhanced by soil nitrogen management—provides a viable strategy for augmenting the carbon sequestration capacity of managed forests through targeted microbiome regulation.

## Simple Summary

Forest soils store vast amounts of carbon, yet the relationship between carbon-fixing microbes and stand age remains unclear. We examined RubisCO-harboring microbes—key players in CO_2_ fixation—across a 70-year chronosequence of *Picea asperata* plantations in Southwest China. Microbial α-diversity decreased progressively with stand age, a trend primarily linked to soil nitrogen content. In contrast, community structure shifted markedly in response to soil pH rather than nitrogen and exhibited a threshold-dependent sensitivity, with sharp shifts occurring within a narrow pH range of 0.5–1.0 units. Our findings suggest that managing soil pH and nitrogen in plantations may help sustain microbial carbon fixation.

## 1. Introduction

Soil represents the largest terrestrial organic carbon pool [1]. Within the forest ecosystem, soil organic carbon (SOC) is predominantly stored in the upper 1 m of the soil profile, accounting for approximately 40% of the global terrestrial soil carbon pool [2]. Shifts in SOC storage reflect a net balance between ecosystem carbon sink and source and are intrinsically coupled to fluctuations in atmospheric carbon dioxide (CO_2_) concentrations. Afforestation has been widely advocated as a strategy to expand forest coverage and enhance terrestrial carbon sequestration, thereby mitigating anthropogenic CO_2_ emissions. However, while afforestation demonstrably augments ecosystem carbon sink through elevated vegetation biomass, the mechanisms governing its impact on SOC storage are yet to be determined [3,4,5]. Empirical evidence shows that afforestation exerts divergent effects on SOC storage contingent upon initial soil carbon status, increasing SOC in carbon-poor soils but reducing it in carbon-rich soils [6]. Moreover, the efficacy of afforestation is also modulated by soil nitrogen (N) availability. Under low-soil-N conditions, afforestation substantially enhances both vegetation biomass and SOC content, while under high-soil-N availability, increases in the ecosystem’s carbon uptake are offset by SOC losses, effectively transitioning the soil from a net carbon sink to a net carbon source [7]. These findings suggest that the impact of afforestation on SOC storage may not only be mediated by vegetation and edaphic conditions, such as nutrient status, but also likely linked to microbially driven carbon inputs and outputs [8]. However, to date, even the fundamental successional dynamics of soil autotrophic carbon-fixing microbial communities and associated key drivers after afforestation remain largely unexplored.

The contribution of afforestation to SOC storage is primarily manifested through vegetation biomass accumulation, litter input, and rhizodeposition—all of which derive from photosynthetically fixed CO_2_ [9,10]. In addition to plant-driven processes, soil microbes also assimilate atmospheric CO_2_ into cellular biomass via carbon fixation pathways, thereby participating in SOC accumulation [11]. Reported rates of microbial CO_2_ fixation in forest soils range from 1.2% to 3.9% of microbial respiration [12] and can reach up to 16% in tundra soils [13]. Notably, a recent study estimated that approximately 26% of the enhanced carbon sink following afforestation originates from SOC accumulation [7]. Hence, microbially mediated CO_2_ fixation represents an important regulatory process governing SOC accumulation. Microbial CO_2_ fixation is driven by phylogenetically diverse autotrophic taxa like *Euryarchaeota*, *Crenarchaeota*, some *Proteobacteria* [14,15] and *Actinobacteria* [16], and a number of green sulfur bacteria [17]—each employing distinct carbon fixation pathways. To date, at least six autotrophic CO_2_ fixation pathways have been identified [18], but the Calvin–Benson–Bassham (CBB) cycle is still the most predominant and widely distributed pathway among autotrophic microbes [8]. The key enzyme of this pathway is ribulose-1,5-bisphosphate carboxylase/oxygenase (RubisCO), which catalyzes the initial CO_2_ fixation reaction of the CBB cycle and exists in four distinct forms: Forms I, II, III, and IV [19]. Among these, several encoding genes such as *cbbL* and *cbbM* have been widely adopted as phylogenetic markers for characterizing autotrophic carbon-fixing microbes [8,20,21]. Given the importance of the CBB cycle and the established utility of RubisCO marker genes, elucidating how carbon-fixing microbial communities shift across stand-age chronosequence is an important step toward understanding microbially mediated carbon dynamics in plantation ecosystems, though this dimension remains poorly constrained.

Stand age is a key determinant of SOC dynamics in plantation forests. A recent study showed that the relative abundance of some potentially carbon-fixing microbial taxa (e.g., *Euryarchaeota*) declined with increasing stand age in spruce plantation soils [22]. In addition, soil nutrient availability, pH, and mean annual precipitation have also been shown to shape soil carbon-fixing microbial communities [23,24], suggesting that both stand age and environmental conditions impact community composition, thereby regulating CO_2_ fixation and SOC storage. However, to date, studies on soil microbial communities in plantation forests have primarily focused on microbial biomass [25], as well as total soil bacterial [26] and fungal communities [27], whereas those explicitly targeting carbon-fixing microbes across the stand-age chronosequence still remain comparatively limited.

In this study, we selected a *Picea asperata* plantation chronosequence in Miyaluo, Lixian, Sichuan Province, Southwest China, as a model system to investigate RubisCO-harboring microbial communities. By integrating metagenomic sequencing, bioinformatics analyses, and multivariate ecological statistics, we aim to address two scientific questions: (i) How do the α-diversity and community composition of RubisCO-harboring microbes shift across the stand-age chronosequence in a *Picea asperata* plantation? (ii) Which edaphic factors predominantly drive the observed shifts in these RubisCO-harboring microbial communities? This study provides a theoretical foundation and novel insights for devising strategies to enhance soil carbon sequestration in plantation forests through targeted microbiome management.

## 2. Materials and Methods

### 2.1. Site Description

This study was conducted in a *Picea asperata* plantation ecosystem located in Miyaluo, Lixian, Sichuan Province, Southwest China (102°41′–102°51′ E, 31°37′–31°49′ N). The study area spans elevations ranging from 2800 to 3400 m above sea level, with mean annual precipitation ranging from 600 to 1100 m and mean annual temperature at approximately 8.9 °C. Soils in the study area are classified as typical brown forest soils [28]. By using a space-for-time substitution approach, four *Picea asperata*-dominated stands of contrasting ages (20, 40, 50, and 70 years old) were selected in August 2024, representing distinct restoration stages following the clear-cutting of natural forests. The understory vegetation in the study area is primarily composed of *Festuca ovina*, *Deyeuxia arundinacea*, and *Carex capilliformis* [29].

### 2.2. Soil Sampling

In each study area, eight 10 m × 10 m plots were established with an inter-plot distance exceeding 50 m. Within each plot, six sampling points were randomly selected, and soil samples (0–10 cm depth) were collected using a 5 cm diameter soil auger. The six subsamples from each plot were thoroughly homogenized to yield a single composite sample and immediately transported to the laboratory. In total, 32 composite soil samples were obtained, with each sample being sieved through a 2.0 mm mesh and divided into two portions: one portion was freeze-dried and stored at −20 °C for subsequent DNA extraction and molecular biology experiments, and the other was stored at 4 °C for soil physicochemical analyses.

### 2.3. Soil Property Measurements

Soil moisture content (SMC) was determined by oven-drying soil to a constant mass at 105 °C for 48 h [30]. Soil temperature (ST) was measured in situ using a geothermometer, and soil pH was measured in a soil–water slurry (1:5, *w/v*) with a pH meter. Total organic carbon (TOC) and total nitrogen (TN) contents were quantified using an automated elemental analyzer (Elementar Vario Macro Cube, Langenselbold, Germany), while total phosphorus (TP) content was determined via the HClO_4_–H_2_SO_4_ digestion method [31].

### 2.4. Soil DNA Extraction and Metagenomic Sequencing

Soil microbial genomic DNA was extracted from 0.5 g of fresh soil using a DNeasy PowerSoil Pro Kit (QIAGEN, Hilden, Germany) following the manufacturer’s instructions. DNA concentrations were assessed using a NanoDrop ND-1000 spectrophotometer (NanoDrop Technologies Inc., Wilmington, DE, USA), and only high-quality DNA was subjected to metagenomic sequencing on the DNBSEQ-T7 platform (BGI Genomics, Shenzhen, China), which employed a paired-end 150 bp strategy. A minimum of 10 GB of clean sequences was generated per sample to ensure sufficient coverage and analytical reliability.

### 2.5. Bioinformatic Analysis of Metagenomic Data

Raw sequencing reads were quality-filtered using fastp v0.24.0 [32], whereby reads with a mean Phred quality score of <20, a length of <100 bp, or containing ambiguous N bases were discarded. Retained paired-end reads were assembled into contigs using MEGAHIT v1.2.9 [33], and contigs < 500 bp were subsequently removed with SeqKit v2.9.0 [34]. Assembly statistics were evaluated using QUAST v5.3.0 [35]. Protein-coding genes were predicted from the assembled contigs using Prodigal v2.6.3 [36], and a non-redundant gene catalog was constructed via CD-HIT v4.8.1 [37]. To identify RubisCO-encoding genes, the non-redundant gene set was queried against a RubisCO database [20] (database available at https://github.com/alexanderjaffe/rubisco-genomics, accessed on 20 December 2024) using hmmsearch v3.4 [38]. Hits with an E-value ≤ 1 × 10^−5^ were retained, and the corresponding DNA and deduced amino acid sequences were retrieved using custom Python (v3.10) and R (v4.5.0) scripts. Relative abundances of RubisCO-encoding genes were quantified through mapping quality-filtered reads to the retrieved DNA sequences with Bowtie2 v2.5.4 [39], and Transcripts Per Million (TPM) values were calculated using CoverM v0.7.0 [40]; TPM matrices were merged across all soil samples using csvtk v0.33.0 (https://github.com/shenwei356/csvtk, accessed on 20 December 2024). Taxonomic assignment of RubisCO-encoding sequences was performed by aligning deduced amino acid sequences against the NCBI non-redundant (NR) protein database using DIAMOND v2.1.11 [41], with taxonomic lineage information extracted via TaxonKit v0.18.0 [42]. All computational analyses were executed on a high-performance computing server (AMD EPYC 7Y43 × 2, 192 threads, 1 TB RAM, 24 TB storage).

### 2.6. Statistical Analysis

Differences in soil properties and Shannon–Wiener indices among stand ages were assessed using Dunn’s Kruskal–Wallis multiple comparisons test. Shifts in the soil properties and community composition of RubisCO-harboring microbes across stand ages were visualized using principal component analysis (PCA) based on Euclidean distance and principal coordinates analysis (PCoA) based on Bray–Curtis dissimilarity, respectively. The statistical significance of these shifts was evaluated using the analysis of similarity with Euclidean distance for soil properties and Bray–Curtis dissimilarity for microbial communities. Pairwise differences in community structure between adjacent stand ages, as well as between the 20- and 70-year-old stands, were tested using a Wilcoxon rank-sum test. Shared and unique RubisCO-encoding gene sequences across stand ages were analyzed using a Venn diagram, while the community composition of RubisCO-harboring microbes at the phylum level was visualized using a stacked bar chart. Microbial taxa exhibiting differential abundances among stand ages were identified using linear discriminant analysis effect size (LEfSe), with significance thresholds set at an LDA score of ≥3 and *p* < 0.01. The key drivers of the shifts in Shannon–Wiener indices and differentially abundant taxa along the stand-age chronosequence were determined using Spearman’s rank correlation analysis, while those shaping the community of RubisCO-harboring microbes across the stand-age chronosequence were ascertained using a redundancy analysis (RDA) based on Bray–Curtis dissimilarity. Environmental variables were fitted onto the RDA ordination to estimate their contributions to community variation, and those with substantial contributions were individually regressed against Shannon–Wiener indices and Bray–Curtis dissimilarities. All analyses were implemented in R v4.4.3, with reliance on the R packages mainly including microeco v1.14.0 [43] and ggplot2 v3.5.1 [44]. Unless otherwise specified, all *p*-values were adjusted using the false discovery rate (*FDR*) correction to account for multiple comparisons.

## 3. Results

### 3.1. Shifts in Soil Microhabitat Characteristics Across Stand Age

**Figure 1:**
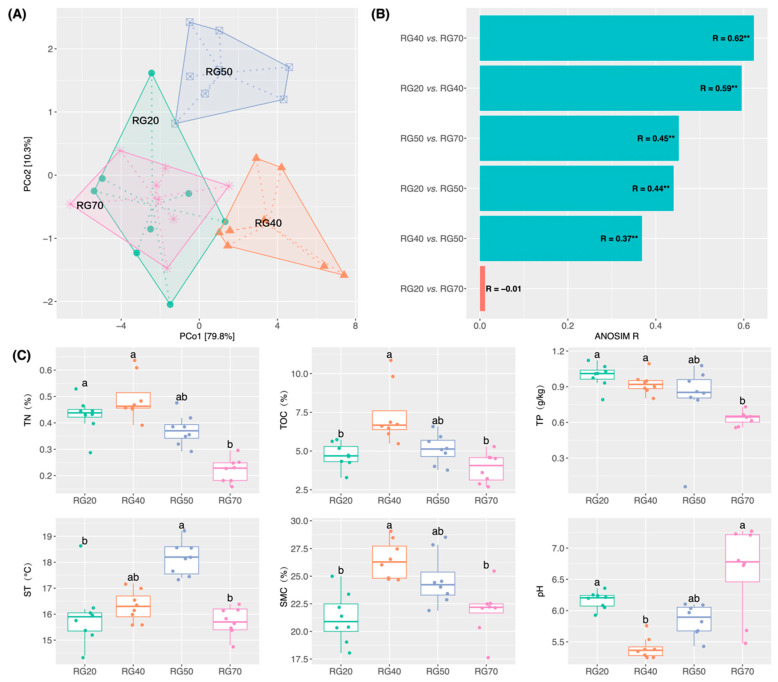
Soil properties across the *Picea asperata* stand-age chronosequence: (**A**) Principal component analysis (PCoA), in which sample points are labeled by stand age. (**B**) Analysis of similarities (ANOSIM) of soil properties. R statistic indicates the effect size, with values approaching 1 denoting greater inter-group differentiation if *p* < 0.05. (**C**) Dunn’s Kruskal–Wallis multiple-comparison test of soil properties. Boxplots with different lowercase letters denote statistically significant differences (*p* < 0.05); groups sharing the same letter are nonsignificant. TN: total nitrogen; TOC: total organic carbon; TP: total phosphorus; ST: soil temperature; SMC: soil moisture content. RG20, RG40, RG50, and RG70 correspond to 20-, 40-, 50-, and 70-year-old plantations, respectively. Asterisks indicate statistical significance at ** *p* < 0.01.

PCoA coupled with the analysis of similarity revealed notable differences (*p* < 0.05) (except 20- vs. 70-year-old stands) in soil microhabitat characteristics among four stand ages (Figure 1A,B). TN, TOC, ST, and SMC showed a unimodal pattern along the stand-age chronosequence, initially increasing and subsequently declining. Specifically, TN, TOC, and SMC peaked at the 40-year-old stand, whereas the lowest values were recorded at the 70-year-old stand (with the exception of SMC) (Figure 1C). ST reached its maximum at the 50-year-old stand and its minimum at the 70-year-old stand, while TP showed a decrease with stand age, although pairwise differences were not all statistically significant. Soil pH declined from the 20-year-old to the 40-year-old stand and then progressively increased through the 50- and 70-year-old stands (Figure 1C).

### 3.2. Shifts in Diversity and Community Structure of RubisCO-Harboring Microbes Across Stand Age

**Figure 2:**
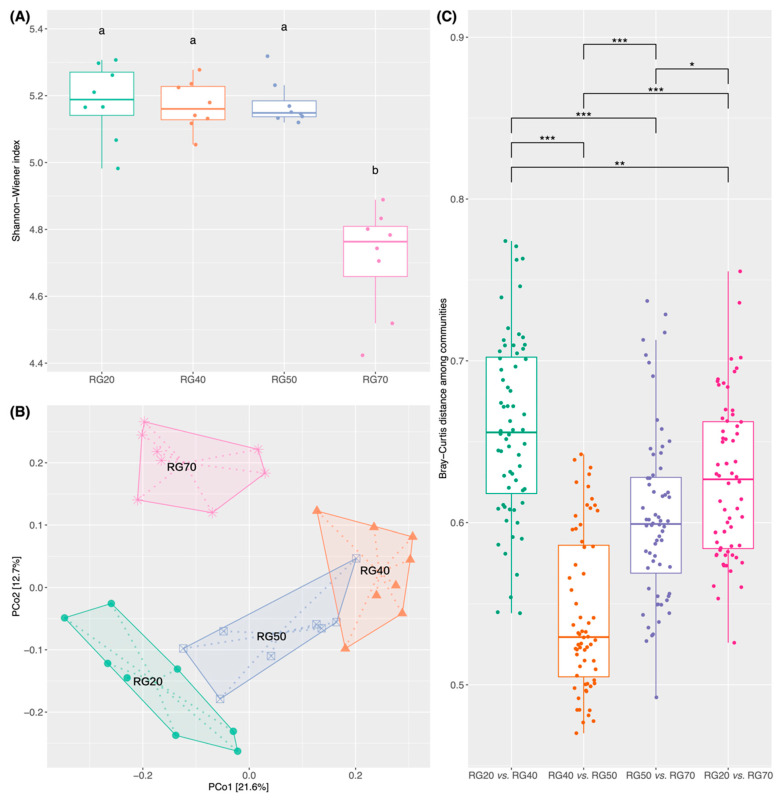
Shifts in the α-diversity and community structure of RubisCO-harboring microbes across the *Picea asperata* stand-age chronosequence: (**A**) A Shannon–Wiener index boxplot, in which the different lowercase letters denote statistically significant differences (*p* < 0.05); groups sharing the same letter are nonsignificant. (**B**) PCoA based on Bray–Curtis dissimilarity, in which sample points are labeled by stand age. (**C**) Pairwise community dissimilarities between adjacent stand ages. RG20, RG40, RG50, and RG70 correspond to 20-, 40-, 50-, and 70-year-old plantations, respectively. Asterisks indicate statistical significance at * *p* < 0.05, ** *p* < 0.01, and *** *p* < 0.001.

A total of 607 RubisCO-encoding gene sequences were identified, comprising 403 Form I, 4 Form III, and 200 Form IV sequences. The Shannon–Wiener index did not differ significantly among 20-, 40-, and 50-year-old stands but decreased markedly in the 70-year-old stand (Figure 2A). PCoA revealed a significant difference in the community structure of RubisCO-harboring microbes across all stand ages (Figure 2B). Pairwise ANOSIM indicated that the greatest compositional dissimilarity occurred between the 20- and 40-year-old stands (R = 0.94; *p* < 0.01), whereas the smallest was observed between the 20- and 50-year-old stands (R = 0.54; *p* < 0.01) (Table 1). Comparisons between adjacent stand ages further showed that dissimilarities between the 20- and 40-year-old stands significantly exceeded those observed between the 40- and 50-year-old and between the 50- and 70-year-old pairs (*p* < 0.001) (Figure 2C). In contrast, dissimilarities between the 40- and 50-year-old stands were significantly lower than those of the other adjacent age pairs (*p* < 0.001) (Figure 2C). Interestingly, the compositional changes from 20 to 40 years exceeded those observed between the 20- and 70-year-old stands (*p* < 0.01) (Figure 2C).

### 3.3. Shifts in Taxonomic Composition of RubisCO-Harboring Microbial Community Across Stand Age

**Figure 3:**
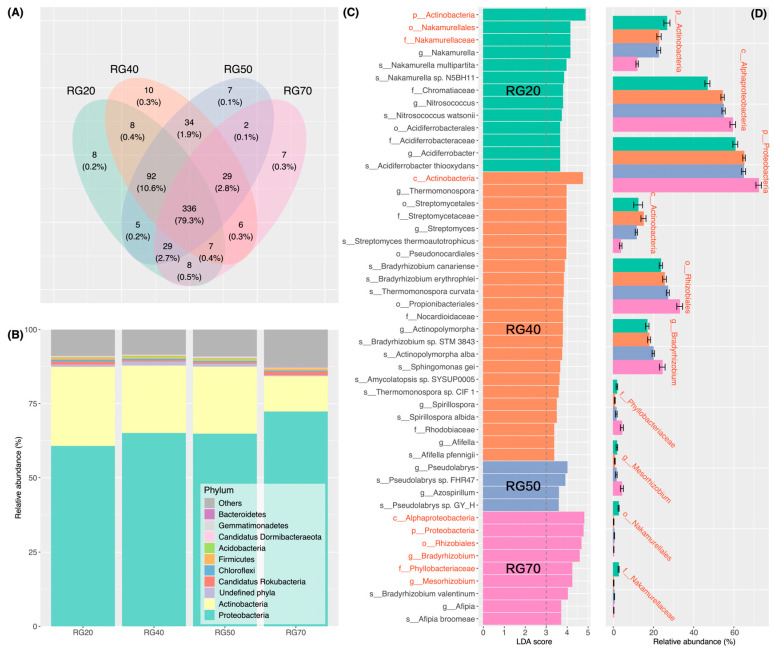
Shifts in the taxonomic composition of RubisCO-harboring communities across the *Picea asperata* stand-age chronosequence: (**A**) Venn diagram revealing shared and unique RubisCO-encoding genes among stand ages. (**B**) Taxonomic composition at the phylum level. “Undefined phyla” represents taxa that cannot be annotated at the phylum level; “Others” represents taxa with low relative abundance. (**C**) LEfSe results identifying 49 taxa with differential abundance across stand ages. Red labels on the *y*-axis indicate the top 10 most differentially abundant taxa, corresponding to the red-labeled taxa in panel (**D**). Text on bars denotes stand age, and bar colors have the same definitions as in panel (**D**). (**D**) Shifts in the top 10 most differentially abundant taxa across the stand age. Error bars denote standard deviation. RG20, RG40, RG50, and RG70 correspond to 20-, 40-, 50-, and 70-year-old plantations, respectively.

The Venn diagram indicated that there were 8, 10, 7, and 7 unique RubisCO-encoding gene sequences in the 20-, 40-, 50-, and 70-year-old stands, respectively, whereas 336 sequences were shared across all stand ages (Figure 3A). Specifically, 8 sequences were shared between the 20- and 40-year-old stands, 34 between the 40- and 50-year-old stands, and only 2 between the 50- and 70-year-old stands (Figure 3A). RubisCO-harboring communities were predominantly composed of *Proteobacteria*, *Actinobacteria*, *Candidatus Rokubacteria*, *Chloroflexi*, *Gemmatimonadetes*, *Firmicutes*, *Bacteroidetes*, and *Acidobacteria* (Figure 3B). With increasing stand age, the relative abundances of *Proteobacteria* increased, whereas those of *Actinobacteria* declined. *Candidatus Rokubacteria* exhibited an initial decrease followed by a subsequent increase, peaking in the 70-year-old stand. The relative abundances of *Chloroflexi* and *Firmicutes* showed minimal variation across the chronosequence, while those of *Acidobacteria* and *Gemmatimonadetes* increased initially and then declined, reaching their lowest values in the 70-year-old stand (Figure 3B). LEfSe results identified 49 differential taxa across the chronosequence, among which *Phyllobacteriaceae* (chemoautotrophs) [45], *Alphaproteobacteria* (chemoautotrophs) [46], *Rhizobiales* (chemoautotrophs) [46], *Bradyrhizobium* (chemoautotrophs) [46], *Mesorhizobium*, and *Proteobacteria* generally increased in relative abundance along the stand-age chronosequence (Figure 3C,D), while *Actinobacteria* (chemoautotrophs) (phylum and class levels) [46], *Nakamurellales*, and *Nakamurellaceae* (chemoheterotrophs) [47] exhibited opposite trends (Figure 3D).

### 3.4. Factors Driving the Succession of RubisCO-Harboring Microbial Communities Across Stand Age

**Figure 4:**
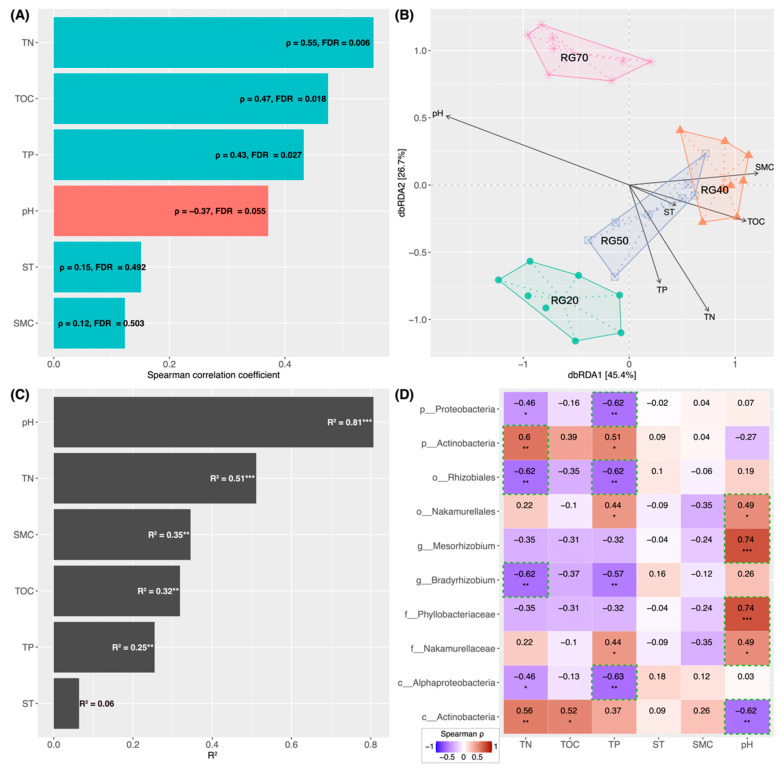
The relationship between soil properties and RubisCO-harboring microbial communities across the *Picea asperata* stand-age chronosequence: (**A**) Spearman’s rank correlations between soil properties and the Shannon–Wiener index. (**B**) The RDA of RubisCO gene-harboring microbial communities based on Bray–Curtis dissimilarities, in which sample points are labeled by stand age and arrows represent soil properties. (**C**) Relative contributions of soil properties to community shifts. R^2^ indicates the proportion of variance explained by each variable; a higher value denotes a greater relative contribution when *p* < 0.05. (**D**) Spearman’s rank correlations between soil properties and the 10 most differentially abundant taxa. Green boxes highlight soil properties with the largest absolute correlation coefficient for each taxon. In panels (**A**,**D**), ρ values denote Spearman’s rank correlation coefficients; a larger absolute value indicates a stronger correlation if *p* < 0.05. TN: total nitrogen; TOC: total organic carbon; TP: total phosphorus; ST: soil temperature; SMC: soil moisture content. Asterisks indicate statistical significance at * *p* < 0.05, ** *p* < 0.01, and *** *p* < 0.001.

**Figure 5:**
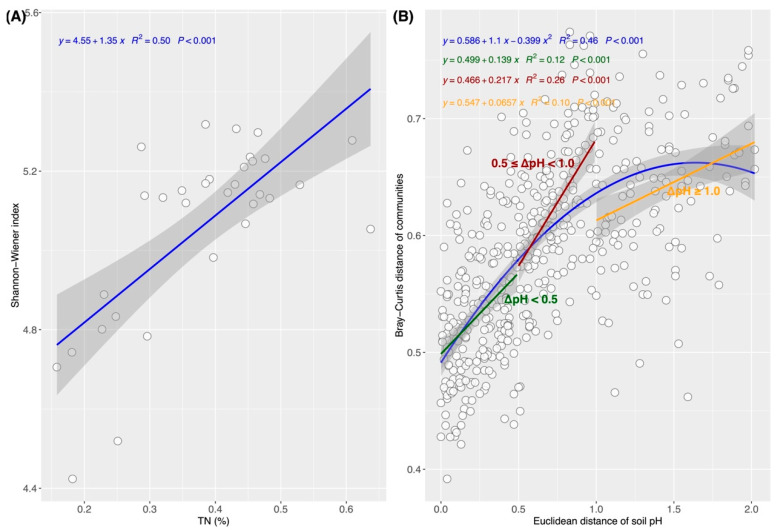
Relationships between the Shannon–Wiener index and soil total nitrogen (TN) and between community dissimilarities and soil pH across the *Picea asperata* stand-age chronosequence: (**A**) A linear regression model for the Shannon–Wiener index and TN. (**B**) Nonlinear regression models for the Euclidean distances of soil pH (ΔpH) and the Bray–Curtis distances of RubisCO-harboring microbial communities. The steepest change rate (slope) was observed within the ΔpH range of 0.5–1.0 units. The shaded area represents the 95% confidence interval.

The Shannon–Wiener index significantly positively correlated with TN, TOC, and TP, whereas no significant correlations were detected with soil pH, ST, or SMC (Figure 4A). Among these, the strongest correlation was observed with TN (Figure 4A), and the Shannon–Wiener index increased linearly with increasing TN content (Figure 5A). RDA identified TN, TOC, TP, SMC, and pH as critical drivers of structural variation in communities across the stand-age chronosequence (Figure 4B), in which soil pH exerted the most pronounced effect on community structure, followed by TN (Figure 4C). Interestingly, the relationship between community dissimilarity and ΔpH was nonlinear, with the steepest change rate occurring within the ΔpH range of 0.5–1.0 units (Figure 5B). Regarding individual taxa, soil pH displayed significant positive correlations with *Phyllobacteriaceae*, *Mesorhizobium*, *Nakamurellales*, and *Nakamurellaceae* but was negatively correlated with *Actinobacteria* (class level), with *Phyllobacteriaceae* and *Mesorhizobium* exhibiting the strongest correlations (ρ = 0.74; *p* < 0.001) (Figure 4D). TN significantly positively correlated with *Actinobacteria* (phylum and class levels) and significantly negatively correlated with *Proteobacteria*, *Rhizobiales*, *Bradyrhizobium*, and *Alphaproteobacteria*; the highest positive correlation was observed with *Actinobacteria* (phylum level) (ρ = 0.60; *p* < 0.01), while the strongest negative correlations were detected with *Rhizobiales* and *Bradyrhizobium* (ρ = −0.62; *p* < 0.01). TP markedly negatively correlated with *Proteobacteria*, *Rhizobiales*, *Bradyrhizobium*, and *Alphaproteobacteria* and positively correlated with *Actinobacteria* (phylum level), *Nakamurellaceae*, and *Nakamurellales*; the highest positive and negative correlation coefficients were recorded for *Actinobacteria* (phylum level) (ρ = 0.51; *p* < 0.05) and *Alphaproteobacteria* (ρ = −0.63; *p* < 0.01), respectively. TOC significantly positively correlated with *Actinobacteria* (class level), while SMC and ST showed no significant correlations with these microbial taxa (Figure 4D).

## 4. Discussion

### 4.1. Stand-Age-Dependent Shifts in Soil Microhabitat Characteristics

**Figure 6:**
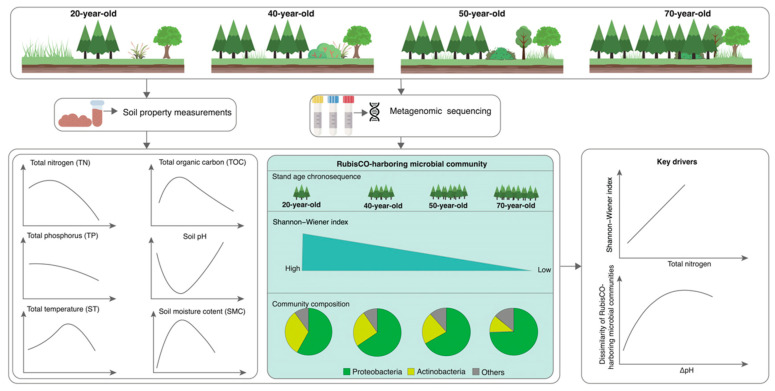
Overview of the primary conclusions drawn from the present study.

Our results demonstrate that soil properties exhibited pronounced directional changes with stand age (Figure 1A,B), underscoring the role of stand development in reshaping soil microhabitats. Specifically, soil nutrient contents (TN, TP, and TOC) showed an overall declining trend (Figure 1C), consistent with the general pattern of nutrient depletion observed during long-term succession in subalpine spruce forests [22,48]. This could be attributed to at least two underlying mechanisms: first, *Picea asperata* plantations are characterized by high planting density and monoculture, and tree growth imposes substantial nutrient demands on soils [49]; second, nutrient inputs to the topsoil in subalpine coniferous forests are primarily derived from litter decomposition [50]. Although litter accumulation increases with stand age, canopy closure concurrently intensifies, reducing understory light availability and further slowing down the decomposition of coniferous litter—which is inherently recalcitrant—thereby impeding nutrient return to soils [50,51]. Furthermore, ST and SMC exhibited a unimodal pattern with stand age (Figure 1C), implying that middle-aged *Picea asperata* stands (i.e., 40- and 50-year-old) may be conducive to maintaining favorable soil hydrothermal conditions. This is likely attributable to the combined impacts of canopy architecture, root activity, litter accumulation, and microclimate [52]. Soil pH displayed an initial decrease followed by a gradual increase, with low values in the 40- and 50-year-old stands (Figure 1C), a trend consistent with a previous study [22]. The decrease in pH could be explained by the relatively higher soil temperature and moisture content in these middle-aged stands, which stimulate microbial activity and litter decomposition, leading to the release of organic acids (e.g., humic acids and carboxylic acids) and a consequent decline in soil pH. This is supported by the high soil organic carbon content observed in the 40- and 50-year-old stands (Figure 1C). Collectively, these findings indicate that soil microhabitats synchronously shift with the secondary succession of *Picea asperata* plantations, showing a pronounced stand-age dependence (Figure 6). We note, however, that the space-for-time design cannot fully exclude the possibility that some of the observed soil differences reflect inherent site heterogeneity or historical contingencies predating plantation establishment.

### 4.2. Marked Shifts in the Diversity and Community of RubisCO-Harboring Microbes with Stand Age

We observed a progressive decline in the α-diversity of RubisCO-harboring microbes with stand age (Figure 6), with the Shannon–Wiener index values at the 70-year-old stand being significantly lower than those at younger stand ages (Figure 2A). The reduction in α-diversity in the oldest stand may signal a diminished genetic potential for CBB-dependent carbon fixation, though more direct evidence is required to confirm this. Notably, the decrease in TOC in the 70-year-old stand raises the possibility that reduced α-diversity of RubisCO-harboring microbes may reduce soil carbon accumulation, though this is speculative in the absence of flux-based evidence. We also found that RubisCO-harboring microbial communities markedly shifted with stand age (Figure 2B; Table 1), mirroring the successions previously reported for total soil bacterial communities [53,54]. Additionally, the number of RubisCO genes shared between the 50- and 70-year-old stands was exceptionally low (Figure 3A). These findings suggest that the diversity loss of RubisCO-harboring microbes in the 70-year-old soil may be closely linked to the pronounced community composition turnover occurring at this successional stage.

RubisCO-harboring communities were primarily composed of *Proteobacteria* and *Actinobacteriota* (Figure 3B). The relative abundances of *Proteobacteria* increased with stand age, whereas those of *Actinobacteriota* declined (Figure 3D), a trend in line with previous findings on soil bacterial succession [54,55]. In this study, *Proteobacteria* were mainly represented by *Alphaproteobacteria* and *Rhizobiales*, both of which were enriched by increased stand age (Figure 3D). Members of *Rhizobiales*, such as *Bradyrhizobium* and *Mesorhizobium*, are known to facilitate plant–microbe interactions via symbiotic nitrogen fixation [56,57]. The metabolic versatility of these taxa enables them to acquire nitrogen via N_2_ fixation in low-nitrogen environments while simultaneously fixing CO_2_ through the CBB cycle, thereby adopting a nitrogen–carbon synergistic strategy [58] that may confer an adaptive advantage under the oligotrophic conditions characterizing the 70-year-old plantation. Conversely, the predominance of *Actinobacteriota* in the 20-, 40-, and 50-year-old stands may be an indicator of their high efficiency in litter decomposition, as *Actinobacteriota* are known to accelerate carbon mineralization by secreting cellulases and lignin enzymes [59]. In the present study, *Actinobacteriota* were dominantly composed of the class *Actinobacteria*, whose relative abundance closely paralleled the shifting pattern of TOC, peaking at the 40-year-old stand and reaching a minimum at the 70-year-old stand (Figure 1C and Figure 3D). Such a congruence suggests that soil organic carbon dynamics in *Picea asperata* plantations may be regulated by both autotrophic carbon fixation and the roles of *Actinobacteria* in organic matter decomposition, though more direct evidence is required.

### 4.3. Shifts in Soil pH and Total Nitrogen Content Drive the Succession of RubisCO-Harboring Communities

Our results show that TN exerted a stronger influence on the α-diversity of RubisCO-harboring microbes, whereas soil pH contributed more substantially to the shifts in community structure (Figure 6). The Shannon–Wiener index increased with increasing TN (Figure 4A and Figure 5A), a finding consistent with previous reports that soil total nitrogen is key in shaping prokaryotic communities in spruce forest soils [60,61]. In this study, TN generally declined with stand ages, which may have imposed nitrogen limitations on RubisCO-harboring microbes, thereby contributing to the decline in α-diversity. This suggests that maintaining or enhancing soil nitrogen availability could help preserve the diversity of carbon-fixing microbes. Numerous studies have demonstrated that soil pH exerts a strong impact on the soil microbial community [60,61,62]. In agreement with these findings, our results identified pH as the key factor driving shifts in community composition along the stand-age chronosequence (Figure 4B,C), but soil pH likely indirectly modulates community through its influence on soil physicochemical properties. Furthermore, since microbes maintain a near-neutral intracellular pH [63], their disparate tolerances to variations in extracellular pH also likely generate physiological stresses that directly structure the communities [64]. Interestingly, we observed a threshold-dependent relationship between community dissimilarities and ΔpH, with a pronounced impact on community structure when the pH difference ranged from 0.5 to 1 units and attenuation when it exceeded 1 unit (Figure 5B). Such a finding implies that the response of RubisCO-harboring microbial communities to soil pH shifts across the stand-age chronosequence is nonlinear and threshold-dependent.

## 5. Conclusions

By examining the successional dynamics of RubisCO-harboring microbial communities across a stand-age chronosequence in *Picea asperata* plantations, we demonstrate that stand age significantly regulates the diversity and composition of these functionally critical microbes via alterations in soil environmental conditions. Soil pH emerged as the predominant factor shaping community structure, while total nitrogen content was the key driver of α-diversity. The decrease in diversity and the compositional shifts observed in older stands may indicate a potential reduction in microbial carbon fixation capability. Overall, these findings underscore the important role of soil physicochemical properties in regulating microbial carbon sequestration processes in plantation ecosystems through the lens of community ecology. Nevertheless, several limitations warrant consideration: First, the space-for-time substitution approach used herein may be confounded by inherent site heterogeneity and historical contingencies, and the observed patterns in this study cannot be unambiguously attributed to stand age alone. Hence, long-term monitoring or experimental manipulations are necessary to validate the inferred successional patterns. Second, our exclusive focus on the CBB cycle overlooks alternative CO_2_ fixation pathways (e.g., the rTCA cycle and the Wood–Ljungdahl pathway) that may contribute substantially to soil carbon dynamics [11,18]. Finally, the conclusions in this study are based on correlative evidence rather than causal inference, highlighting a need for targeted manipulations (e.g., nitrogen addition or pH adjustment) to establish mechanistic links between soil properties and community responses. Addressing these gaps will be essential for advancing a comprehensive and mechanistic understanding of microbial carbon fixation in forest soil management.

## Figures and Tables

**Table 1:** Analysis of similarity (ANOSIM) for RubisCO-harboring microbial communities.

Comparison	R Value	*p*-Value	*FDR* Value
**RG20 vs. RG40**	**0.94**	**0.001**	**0.002**
RG20 vs. RG50	0.54	0.002	0.0024
RG20 vs. RG70	0.75	0.001	0.002
RG40 vs. RG50	0.62	0.001	0.002
RG40 vs. RG70	0.85	0.002	0.0024
RG50 vs. RG70	0.70	0.003	0.003

**Note:** RG20, RG40, RG50, and RG70 correspond to 20-, 40-, 50-, and 70-year-old *Picea asperata* plantations, respectively. The highest R values are highlighted in bold. *FDR*: false discovery rate.

## Data Availability

Data will be made available upon request.
